# Institutional gap and enterprise behavior: Evidence from China

**DOI:** 10.1371/journal.pone.0297455

**Published:** 2024-03-01

**Authors:** Meijie Yao, Wenxue Wang, Li Rao

**Affiliations:** 1 School of Business Administration, Chongqing Technology and Business University, Chongqing, PRC; 2 School of Finance, Shandong Technology and Business University, Yantai, PRC; Lingnan University, The University of HongKong, HONG KONG

## Abstract

Alternative institutions have played a significant role in the difficult and tenacious development process of private enterprises in recent decades. the paper applied 306 listed companies’ data from January 2012 to December 2022 to investigate the relationship between institutional gap and enterprise behavior. Based on the identified, classified, and explained formal and alternative institutions, it proposed the concept of ’institutional gap’ to infer the overlapped content of these two states. Concluding from the empirical results, Chinese private enterprises that introduced state-owned equity outperformance in management, financing and entering industries with high barriers. Moreover, the channel of state-owned equity introduction is compared with entrepreneurs’ political participation. Although introducing state-owned equity is a crucial approach, the establishment and improvement of relevant legal systems is suggested, such as formal regulations and laws. The government should deepen the reform of the economic system to improve the business environment, so private enterprises consciously rely on market mechanisms to allocate resources efficiently and rationally, instead of actively seeking connections with the government to obtain benefits. The supervisory role of checks and balances shareholders should be exerted to the greatest extent to maintain and increase the value of state-owned assets, rather than allowing the controlling private shareholders to use them as resources acquired political capital.

## 1 Introduction

The rapid economic growth recent decade in China has gained the world’s attention. Undoubtedly, private enterprises contributed in multiple aspects. According to the data from the State Administration for Market Regulation (SAMR) of China, the private sector has contributed more than fifty per cent of China’s GDP growth, created ninety per cent of employment, and undertaken more than half of tax revenue payment. In one word, the private enterprises have progressed in all-around results and great achievements although it is a rough journey for the group. The corporate-related policies, laws, and official systems tend to support and assist state-owned enterprises, while the private groups have to explore suitable and effective business development models and strategies to compete with state-owned counterparts. In the various imperfect systems, private enterprises apply the ‘political connections’ strategy to gain management purposes of reducing business risks, improving performance, and achieving rapid growth [1–5]. On the other hand, the government is willing to step into private enterprise operation management through ‘rent-seeking’ [6–8]. Allen et al. [9] conducted a series of empirical studies on the economic and social development of Asia-Pacific countries and regions, in which the famous theory of “alternative institutions” was put forward and the relative effect was confirmed. Therefore, the paper summarizes the ‘political connections’ strategy and ‘rent-seeking’ phenomenon as ‘alternative institution’. The most impressive concept the paper proposed is the ‘institutional gap’, which describes the non-overlapped area between ‘formal institution’ and ‘alternative institution’.

The financial development and economic growth in China have been achieved under weak legal institutions [10]. Socialist countries based on public ownership have formal institutional protection for national property rights. The formal system for the protection of private property rights needs to be improved. State-owned enterprises have inherent advantages, and private enterprises are in virtue of substitution supports. Based on this social environment, the introduction of state-owned shares to private enterprises controlled by private property rights can be regarded as some kind of alternative system to the formal protection system, as well as the participation of private entrepreneurs in politics. These are the ‘alternative institutions’ compared with the legislation specially established to protect state-owned property. To avoid the imperfect protection of private property rights by business-related laws and regulations, companies actively seek protection and even improve their operations through ‘alternative institutions’. The two most commonly used ‘alternative institutions’ are introducing state-owned equity and CEO participation in politics. In the paper, we ask whether the alternative system can be used as a supplement to the ‘formal institution’, especially when we focus on the institutional gap factors–introducing state-owned. Further, we investigate how it works and the various effects result in firm management of introducing state-owned shares. Finally, we compare the impact of introducing state-owned shares and political participation on corporate performance.

There are several other related previous research on formal institutions and alternative institutions [11–14]. However, the empirical results of the relationship between the alternative institutions and private enterprises’ performance are not consistent. An et al. [15] propose that enforced political obedience is not associated with market performance. Fan et al. [16] proved CEOs holding political connections are related to their firm’s stock return underperformances. On the other hand, studies provide evidence that political connections boost corporate value [17], and higher return and outperformance [18]. Further, private companies gain protection through the connection in China called ‘guanxi’ [2]. Also, the bank system shows the dynamics above described that politically connected banks are easier to attract deposits than their counterparts [19].

However, the conceptions of formal and informal institutions are vague under some business management circumstances. Therefore, we proposed the phrase ‘alternative institutions’ to represent the institutions excluded by formal institutions. In the shed light of Allen et al. [20], during China’s economic transformation and rapid economic growth period (1980–2010), the contribution rate of its legal system to economic development was extremely low, and the “alternative institutions” generated by economic reality became the main force driving the efficient operation of the market. These “alternative institutions” include the trading participants’ reputation, social status, and trust to settle disputes within the institutions. The research of Allen et al. [20] undoubtedly captures the key points of institutions in developing countries. We apply the institutional gap to further define the uncovered concept in alternative institution but outside the formal institution.

Based on the previous we discussed, we proposed the research questions below:

**Q1**: Do private enterprises outperformance through introducing state-owned equity compared with those single shareholder private companies?
**Q2**: Does introducing state-owned equity assist private enterprises in entering industries with high barriers?
**Q3**: Do the private enterprises introducing state-owned equity obtain finance support and bank loans more easily than their counterparts with single ownership?
**Q4**: Compared with entrepreneurs’ political participation, do private enterprises introducing state-owned equity exhibit advantages in management performance, enter barrier industry, or gain financial support?

The contributions are summarized as follows. First, the concept of ‘alternative institutions’ is the first time to propose and use in corporate finance field research. It provides a clear demarcation for institutions’ impact on corporate finance. Further, institutional gap is measured as private enterprises introducing state-owned equity, and relative enterprise behaviour is investigated. Second, the paper provides reliable evidence that private enterprises construct protection mechanisms and improve their competitive ability via alternative institutions–introducing state-owned equity. As the traditional way to make political connections, entrepreneurs’ political participation is contrasted with state-owned equity introducing. Therefore, the third contribution is we confirmed state-owned equity introducing mechanism has a stronger effect compared with another political connection method.

The paper is structured as follows. The second part reviews the relevant literature and states the innovation of the study. The third section presents the relative theory framework and proposes research hypotheses. The fourth part illustrates the research design and explains the methodology. The fifth section demonstrates the empirical results. The last section concludes the paper.

## 2 Literature review and study innovation

With the annual double-digit growth in GDP and the substantial improvement of people’s living standards since the reform and opening-up policy in 1978 in China, the institutional environment is still not optimistic. To be explicitly, it is featured an imperfect market system, unequal market entity status, enormous economic barriers, unfriendly financing environment for small and medium-sized enterprises, and difficulty in ordered competition [21–23].

In one region or under similar social and economic backgrounds, several relevant research sheds light on this field. Fan et al. [16] conducted a study on Chinese companies. It concludes that it is an efficient way to have a political connection for a company CEO who works for the government or the military. Nys et al. [19] and Gray and Harymawan [24] supported that the political connection of companies is a relatively common phenomenon, and it is consistent results in both developed and developing countries. Choi et al. [25] found that private enterprises rely on using interpersonal networks to gain business development instead of actively obtaining resources or developing strategic alliances through the market. Xin and Pearce [2] produced the consistent results that the priority of private enterprises in cooperate strategy is to build and maintain the relationship with the government compared with state-owned enterprises. Establishing and consolidating the relationship is helpful to gain the protection and support that is difficult to obtain from formal institutions. According to the research from Faccio [17] on Southeast Asian countries such as Indonesia, the political strategies of listed companies are mainly implemented through establishing personal connections with senior government officials. It analyzed more than 20,000 listed companies in 47 countries and regions to conclude that politically connected companies are more prominent in countries with obvious restrictions on foreign capital entry, serious corruption, and opaque systems. The party membership status of private entrepreneurs helps enterprises obtain financial resources to improve company performance. The above studies limit the political connections of Chinese private enterprises to the political participation of company founders or executives (or key members of the board of directors) or the relationship between company executives and government officials [3]. To summarise, there are a large number of studies on the influence of entrepreneurs’ political participation on incorporate value [26–29], financing [27, 30], financial performance [16, 31, 32], investor protection [33], and protection on intellectual property [34].

The above research we discussed has not discussed the effect of state-owned equity on private enterprise management. In the shed light of Allen et al. [20], the ‘alternative institutions’ concept was put forward and it starts a new era and provides the potential mechanism between commencing development and the institutions outside the legal system. Introducing state-owned equity is a representative way to range business management via establishing alternative institutions. A series of studies we should mention here which combine legal, societal and business operations to discuss issues related to corporate finance. An et al. [10] confirm the relationship between alternative institutions and finance development in China, such as incentives, reputation and relationships. Constraints on bureaucrats’ misconduct induce positive market reactions, especially to private firms [15]. To be impressed, the research combines social development contexts such as epidemic and colonial history with financial development [11, 14]. Another study by An [12] proposes in male favor countries, firms with the majority workers making up with females are disadvantaged in trade credit access. Trust improves credit use for companies facing difficulties gaining financing through formal platforms [14]. Several papers discussed the alternative institutions in related finance fields as well. The study conducts the influence of managerial accountability and sustainable reporting compliance on revenue reported by public institutions in Romania [35]. Grigorescu et al. [36] contribute in suggestions on incorporate sustainability development. Munteanu et al. [37] present the impact on crypto assets of political instability, wars, and pandemics.

## 3 Theory framework and hypotheses development

Chinese private enterprises have grown in an unfavourable institutional environment, while the private firms have greatly contributed to rapid development. Institutional unfriendly issues cause a series of problems for private enterprises, such as ownership dilution, financing constraints, industry barriers and so on. Under this circumstance, the group resort to another set of market operation mechanisms to seek growth and even survive [33]. In shed light of alternative institutions research by An et al. [11], An [12], Xu et al. [13] and An et al. [14], introducing state-owned equity is recognized as an alternative institution for private enterprise construct development strategy. It protects private firms’ rights and interests, reduces business risks, improves company performance, is a booster to enter barrier industry, effectively obtains finance support and bank loans, and so on. In the paper, we test the role of introducing state-owned equity as an ‘alternative institution’ in private enterprise cooperation.

### 3.1 The role of introducing state-owned equity as ‘alternative institutions’ for private enterprises in multi-aspects

State-owned shares are those formed by state-owned assets invested in companies by departments or institutions with the right to invest on behalf of the state. They are generally held by departments or institutions authorized by the State Council, or by departments or institutions authorized by local governments according to the decisions of the State Council. The natural connection of state-owned equity to the government makes it more advantageous in terms of industry expansion, resource acquisition, and property rights protection, and easier to access various preferential policies and treatment. However, in China’s current economic system, it is undeniable that state-owned equity has inherent advantages in business strategies such as property rights protection, resource acquisition, and industry expansion. The introduction of state-owned equity is an important channel for private enterprises to establish political connections, and finally achieve comprehensive development. As regards, the protection and support from introducing state-owned equity, the paper considers it in triple aspects–business performance, barrier industry entering, and financing advantage. It is the logic for the following explanations.

#### 3.1.1 The role of introducing state-owned equity in business performance

The main purpose of introducing state-owned equity is to build a symbiotic relationship with the government, which would be supportive and helpful to private enterprises whether in their survival or development. The advantages are summarised in four aspects. First, the introduction of state-owned equity is beneficial to private companies knowing regional and even national development plans in advance, to make strategic arrangements before the market action. Seizing the opportunity and occupying the market early is a crucial step for enterprise development. Meanwhile, understanding regional development plans, private companies are also willing to participate in government development schedules. It provides an efficient approach for enterprises to obtain preferential treatment and policy orientation. Third, industries with policy tendencies and government support are prone to have industry barriers, and companies with natural ties to the government are relatively more likely to develop in such fields. Moreover, during the business bidding process, companies with government endorsement are more likely to be trusted and selected as business partners. Therefore, we conclude the fourth point that private companies introducing state-owned equity are attractive because of financial situation, reputation, resource channel and so on. The primary reason is that state-owned equity provides brand effect, and it is proof of the company’s strength.

The relevant research shows that under the market economy system in China’s transition period, private-controlled listed companies whose state-owned shares do not occupy the controlling position. The main role of directors representing state-owned shareholders is not only to supervise the opportunistic behavior of managers, but also to reflect a certain degree of connection of the private enterprises with government departments, offset the negative effects related to the economic system environment for the companies [38], and draw external resource support for the companies [39]. Based on the above analysis, Hypothesis 1 is proposed:

**H1.** *Ceteris paribus*, private enterprises outperformance through introducing state-owned equity compared with those single shareholder private companies.

#### 3.1.2 The role of introducing state-owned equity in barrier industry entering

Based on the characteristics of the Chinese economy, the introduction of state-owned shares into equity means that private enterprises have the blood and nature of some state-owned enterprises. At the same time, we can also think of this as a symbiotic relationship.

From the perspective of industry entry, on the one hand, entry into any industry requires approval from relevant government departments and support from registration and other processes. On the other hand, industries such as petroleum, telecommunications, and finance are all dominated by the state and are mainly engaged by state-owned enterprises and central enterprises. Even some monopolistic industries related to the lifeblood of the country require strict review by specialized approval agencies and must comply with the provisions of laws and regulations. Enterprises that introduce state-owned equity are more likely to be trusted by the government when entering barrier industries. Unlike in developed countries, cultivating good relationships with the government is an important business strategy for private companies in industries with entry barriers [40]. The existence of this relationship is not only supportive at the corporate development level but also beneficial in terms of the entry barrier industry. Especially in relatively economically underdeveloped regions, the regulation, police and system of private firm protection are imperfect. Therefore, the connection with the government created through the introduction of state-owned shares is a symbol of strength and status. At the same time, in crucial industries that are related to the lifeline of the country’s survival and development, companies that are already building a relationship to the government are more trustworthy. Further, good cooperation experience majority likely promote further business development.

**H2.** *Ceteris paribus*, the effect of introducing state-owned equity into private-controlled companies on entering barrier industry.

#### 3.1.3 The role of introducing state-owned equity in financing advantage

In the process of China’s economic and social transformation, private enterprises face more difficulties in obtaining financing than state-owned enterprises. With the increase in private enterprises’ investment scale, their development will rely more on external financing [31], which will urge them to seek other channels in the institutional gap to ease the financing constraints. Accessing quality capital sources by building political connections is a coping strategy employed by many private companies in developing countries with inadequate financial markets. Charumilind et al. [41] conducted a study on Thai firms’ access to long-term loans during the 1997 Asian financial crisis and concluded that those with political connections were more likely to obtain long-term loans and less collateral was required for their loans. Based on the investigation into the corruption cases of provincial and ministerial officials in 23 Chinese provinces, Fan et al. [16] found that the asset-liability ratio of private enterprises associated with corrupt officials dropped sharply and the debt maturity structure was also significantly shortened after the officials were caught. Li et al. [3], taking 2,324 private enterprises in China as the research sample, found that entrepreneurs with CPC (Communist Party of China) membership was more likely to obtain loans from state-owned banks than entrepreneurs without CPC membership. There are a large number of documents showing that the political connection between enterprises and the government is often regarded as an important long-term investment by private enterprises [2, 16, 25, 42].

State-owned equity shares play a relatively direct political connection role at the institutional level in private holding companies. In other words, part of the state-owned equity contained in private enterprises is the link that maintains the "relationship" between private enterprises and the government. The "symbiotic relationship" effect produced by the mixed ownership model of private equity and state-owned equity has a reputation effect at the alternative institutional level that is more accepted and recognized by society. Moreover, the social resources obtained through this association tend to be more. Banks, agencies, other financial institutions, and government departments may feel more reassured psychologically and may act boldly when doing business with these private enterprises than their counterparts. Based on the above analysis, Hypothesis 3 is proposed:

**H3.** *Ceteris paribus*, with other conditions being the same, the introduction of state-owned equity can help private enterprises obtain more financing.

### 3.2 Contrast on state-owned equity introducing and entrepreneurs’ political participation

Gerschenkron [43] discovered that when industrialization started in backward countries, their organizational structure and production structure were very different, and the differences in these characteristics largely resulted from the use of the institutional tools that mature industrial countries did not have. In China’s social institution of market economy, private enterprises are greatly stimulated to establish political relations with the government. Compared with establishing private connections with government officials or entrepreneurs participating in politics, introducing state-owned equity into the equity structure brings a more certain and long-term reputation guarantee. With cross-period and cross-regional credibility, this behaviour is interpreted by outsiders as a stable institutional-level linkage and a deep-rooted linkage of interests. Therefore, when state-owned equity is included in the equity structure of private enterprises, its role should exceed the impact of private entrepreneurs’ political participation on corporate performance. At this time, the dependence of private enterprises on entrepreneurs’ political participation will be reduced. As one of the most important factors of the institutional gap, we believe the introduction of state-owned equity has a significant role compared with entrepreneurs’ political participation (overlapped area between ‘formal institution’ and ‘alternative institution’). Based on the above analysis, Hypothesis 4 is proposed:

**H4.** *Ceteris paribus*, the introduction of state-owned equity into private enterprises, being a more efficient alternative institution compared with entrepreneurs’ political participation, has a significant effect in improving overall performance.

**Fig 1:**
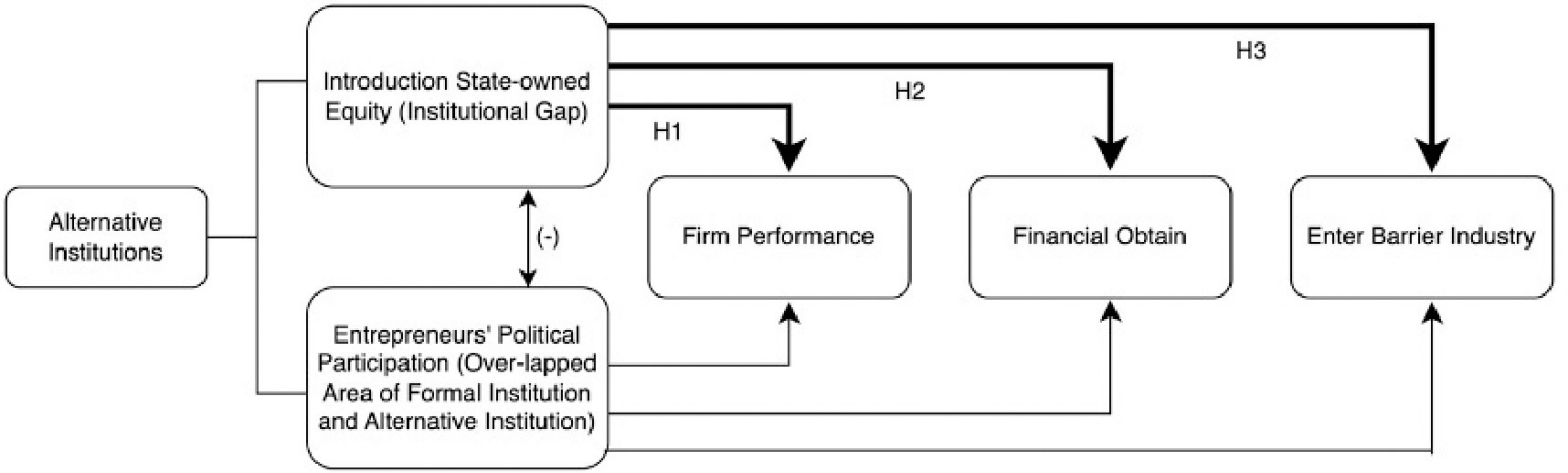
Theoretical framework and hypotheses development.

The relationship of theoretical framework and each hypothesis to be tested in the next part are shown in Fig 1.

## 4 Research design and methodology

### 4.1 Sample selection and data source

The study applied the private-controlled A-share listed companies on Shanghai Stock Exchange and Shenzhen Stock Exchange, because the largest shareholders of private enterprises in these exchange markets have clear private property rights. The equity structures and financial data of the companies were retrieved from the Wind database and CSMAR, and the data concerning the pillar industries of the provinces where the companies are located were collected from official government websites. The sampling period from January 2012 to December 2022, and 306 sample companies and 3227 observations were obtained. The paper determines the actual control chain of each company based on the information of equity structure in the annual reports of listed companies in China. Additionally, the original sample was screened, and the companies 1) with incomplete information disclosure, 2) in the financial service sector, 3) with unknown ultimate shareholder, or 4) identified as abnormal such as those receive special treatment to eliminate unreasonable observations.

### 4.2 Econometric model and variable description

The paper selected the state-owned equity contained in the private-controlled companies to measure the companies’ alternative institution. Alternative institution is defined as the state-owned equity owned by the top ten shareholders of a private company. If the company contains more than one state-owned shareholder, the shares would be combined, and the variables depicting this factor are: 1) whether there is state-owned equity (*SOE*), 2) state-owned equity ratio (*SOER*), 3) state-owned equity background (*SOEB*), and 4) ratio of directors representing state-owned equity on the board of directors (*SOEBR*).

In addition, to compare with the existing research results of political connection, this paper also analyzed the political participation of private entrepreneurs. At present, the primary formal ways for private entrepreneurs to participate in politics include: 1) to enter the People’s Congress or the Chinese People’s Political Consultative Conference (CPPCC) at different levels, 2) to serve in the All-China Federation of Industry and Commerce, Youth Federation, or Women’s Federation, and 3) to join the CPC or a democratic party. In this paper, entrepreneurs’ political participation is specified to the fact that the actual controller/chairman or general manager of the company is elected as a member of People’s Congress or CPPCC. The empirical part is divided according to the principle of corporate governance. To be explicit, whether a company has political identity was firstly determined according to its actual controller. In the robustness test, alternative regression tests were performed with the political identity of a company’s general manager. Table 1 shows the distribution of political connections of the sample companies.

**Table 1:** Distribution of political connections of sample companies.

	With state-owned equity	Without state-owned equity	Total
**Without political participation**	42 (Sample A)	71 (Sample B)	113
**With political participation**	95 (Sample D)	98 (Sample C)	193
**Total**	137	169	306

Table 1 reveals that 137 of the 306 private-controlled listed companies have state-owned shares held by the top ten shareholders, accounting for 44.8%. Among them, 42 companies featured merely by state-owned equity constitute Sample A; 95 companies with both state-owned equity and entrepreneurs’ political participation constitute Sample D; 98 companies featured merely by entrepreneurs’ political participating are Sample C; 71 companies with neither state-owned equity nor entrepreneurs’ political participation are Sample B, accounting for 23% of the total sample. Statistics show that at this stage, it is very common for private-controlled listed companies in China to have political connections.

The econometric model used in the paper is:

$$D_{it}=\alpha_{0}+\alpha_{1}{State}_{it}+\alpha_{2}{Control}_{it}+Year+Industry+\varepsilon_{it}$$

where *D* represents the explained variables, *State* represents the variables related to state-owned equity, *Control* represents a series of variables related to company characteristics and political connection, *i* represents different companies on the cross section, and *t* represents different years. In testing H1 and H3, *D* signifies the company performance variables *ROA* and *ROE*; while in testing H2, it signifies the economic resource variables, namely the proportion of bank loans in total assets (*Loan*) and the entry into industries with barriers (*Barrier*). It should be noted that a series of control variables represented by *Control* vary according to different test purposes of the three hypotheses. When testing H2, considering that a company’s financial situation in the previous year would be evaluated by commercial banks when granting loans, data in year *t*-1 were used for all the explanatory variables. Apart from the general variables related to company characteristics, this paper also defined the dummy variable of whether the main business of the private-controlled company belongs to local pillar industries (*Pillar*) and the variable of ultimate control intensity (*Cintensity*).

In terms of the variables related to company characteristics, those included in testing H1 are: financial leverage (*Leverage*), company total assets (*Tassets*), listing time (*Ltime*), company growth (*Growth*), pillar industry dummy (*Pillar*), and ultimate control intensity (*Cintensity*), as well as the dummy variable of entrepreneurs’ political participation (*Political*). Those included in testing H2 are: company total assets (*Tassets*), profitability (*Profit*), fixed assets (*Fassets*), company growth (*Growth*), listing time (*Ltime*), pillar industry dummy (*Pillar*), ultimate control intensity (*Cintensity*), as well as the dummy variable of entrepreneurs’ political participation (*Political*). To test H3, the interaction variables of (*Political×SOER*) and (*Tassets×SOER*) were added to the above variables.

To control the impact of different years and industries, two dummy variables were introduced in the empirical part of this paper. Specifically, year dummy (*Year*) was set by different years, and industry dummy (*Industry*) was set according to the Guidelines on Industry Classification of Listed Companies issued by China Securities Regulatory Commission. See Table 2 for the definitions of the variables used in this paper.

**Table 2:** Definitions and measurement of variables.

Variable	Symbol	Definition
**State-owned equity variables**		
Whether there is state-owned equity	*SOE*	Dummy. The value is set to 1 if the company’s top ten shareholders include state-owned shareholders; otherwise, the value is set to 0.
State-owned equity ratio	*SOER*	State-owned share capital among the top ten shareholders of the company / Total share capital of the top ten shareholders of the company
State-owned equity background	*SOEB*	The value is set to 1 if the state-owned share capital among the top ten shareholders is held by state-owned institutions at or above the prefectural or municipal level or by central enterprises; the value is set to 0.5 if held by state-owned institutions below the prefectural or municipal level, or by general state-owned legal persons; otherwise, the value is set to 0.
Ratio of state-owned directors on the board	*SOEBR*	Number of directors representing state-owned shareholders / Total number of directors
**Company performance variables**		
Return on assets	*ROA*	Net income / Average total assets
Return on equity	*ROE*	Net income / Shareholders’ equity
**Company resource variables**		
Proportion of bank loans in total assets	*Loan*	(Short-term debt + Long-term debt) / Total assets
Industry barriers	*Barrier*	Dummy. The value is set to 1 if the company has entry into industries with barriers; otherwise, the value is set to 0.
**Company characteristic variables**		
Total assets	*Tassets*	The logarithm of the company’s total assets
Financial leverage	*Leverage*	Total debt / Total assets
Profitability	*Profit*	Earnings before interest and taxes (EBIT) / Total assets
Fixed assets	*Fassets*	Net fixed assets / Total assets
Company growth	*Growth*	(Current year sales revenue − Prior year sales revenue) / Prior year sales revenue
Whether the main business belongs to local pillar industries	*Pillar*	Dummy. The value is set to 1 if belongs to local pillar industries; otherwise, the value is set to 0.
Ultimate control intensity	*Cintensity*	Number of shares held by the ultimate controlling shareholder / Number of shares held by state-owned shareholders among the top ten shareholders of the company
Listing time	*Ltime*	Number of years the company has been listed
**Other political connection variables**		
Whether entrepreneurs participate in politics	*Political*	Dummy. The value is set to 1 when the actual controller or general manager of the company is a member of People’s Congress or CPPCC at or above the prefectural or municipal level; otherwise, the value is set to 0.
Year dummy	*Year*	Dummy. The value is set to 1 when the sample observation is in the year; otherwise, the value is set to 0.
Industry dummy	*Industry*	Dummy. The value is set to 1 when the enterprise is in the industry; otherwise, the value is set to 0.

## 5 Empirical results

### 5.1 Descriptive statistics

Table 3 presents the descriptive statistics of the research variables.

**Table 3:** Descriptive statistics of research variables.

Variable	Observation No.	Minimum	Maximum	Mean	Std. deviation
*SOE*	3227	0	1	0.454	0.381
*SOER*	3227	0.000	0.361	0.163	0.312
*SOEB*	3227	0	1	0.221	0.523
*SOEBR*	3227	0.000	0.229	0.134	0.371
*ROA*	3227	-0.239	0.214	0.055	0.631
*ROE*	3227	0.019	0.531	0.129	0.361
*Loan*	3227	0.351	0.773	0.431	0.728
*Barrier*	3227	0	1	0.215	0.212
*Tassets*	3227	16.964	36.856	21.876	15.23
*Leverage*	3227	0.036	0.753	0.562	0.341
*Profit*	3227	-0.321	0.741	0.037	0.489
*Fassets*	3227	0.114	0.532	0.361	0.157
*Growth*	3227	-1.128	19.415	0.131	8.245
*Pillar*	3227	0	1	0.351	0.289
*Cintensity*	3227	1.057	1.749	1.398	0.831
*Ltime*	3227	1	24	6.731	7.617
*Political*	3227	0	1	0.632	0.374

Table 3 shows that the average *SOE* value is 0.454, indicating that it is common for the private enterprises in the research sample to have state-owned equity, while the average *SOER* value is 0.163. The average value of *Political* is 0.632, implying that nearly two thirds of the private entrepreneurs in the sample are members of People’s Congress or CPPCC at or above the prefectural or municipal level. The average amount of bank loans of private-controlled companies accounts for about 43.1% of the company total assets, which shows that even though Chinese listed companies have other financing channels, their main source of financing is bank loans. Only 21.5% of the sample private companies have entered industries with high barriers (*Barrier*), revealing that private enterprises still face great restrictions in the process of entering industries with high barriers. The standard deviation of *Loan* is large, which indicates that significant differences exist in different companies’ ability to obtain bank loans.

### 5.2 Analysis of regression results

The observation samples in this paper are comprised of cross-sectional data, time-series data, and unbalanced panel data. Because panel data models include fixed effects model and random effects model, and there are great differences between the two in practice, the Hausman test was carried out first. The result shows a p value of 0.0021, so the null hypothesis was rejected. Hence, it can be seen that the econometric model set in this paper has fixed effects, so the fixed effects model was used for regression. In the model estimation, the model standard errors were adjusted for heteroskedasticity and the company observations were adjusted for autocorrelation to obtain accurate t-statistics. In addition, the company resource variable *Barrier* is a dummy, so logistic regression was employed when it was treated as an explained variable.

#### 5.2.1 The impact of political connection resulted from state-owned equity on enterprise performance

Table 4 reports the estimated results of the impact of political connection resulted from state-owned equity on enterprise performance.

**Table 4:** The impact of state-owned equity on the performance of private-controlled enterprises.

Variable	*ROA*			*ROE*		
	(1)	(2)	(3)	(4)	(5)	(6)
*SOE*	0.036***	—	0.027**	0.055***	—	0.056**
	(2.423)		(2.035)	(2.213)		(2.308)
*SOER*	—	0.073***	0.019**	—	0.045**	0.065*
		(2.877)	(2.185)		(2.156)	(1.516)
*Tassets*	0.027	0.054	0.064	0.0731	0.021	0.085
	(1.075)	(1.109)	(1.093)	(1.075)	(1.034)	(0.808)
*Leverage*	0.065	0.096	0.078	0.125	0.076	0.064
	(0.864)	(1.007)	(0.983)	(0.778)	(0.690)	(0.782)
*Growth*	0.026*	0.084*	0.049	0.065*	0.079	0.109*
	(1.543)	(1.473)	(0.834)	(1.503)	(0.661)	(1.512)
*Pillar*	0.094**	0.092***	0.028**	0.038***	0.026**	0.532**
	(2.324)	(3.043)	(2.105)	(2.863)	(2.165)	(1.943)
*Cintensity*	0.059	0.027	0.017	0.207	0.071	0.063
	(0.719)	(0.891)	(0.710)	(0.943)	(0.659)	(0.879)
*Ltime*	-0.041	-0.038	-0.047	-0.073	-0.041	-0.092
	(-1.041)	(-0.910)	(-0.867)	(-0.683)	(-0.969)	(-0.762)
*Political*	—	—	0.013***	—	—	0.063*
			(1.494)			(1.507)
Adjusted- *R*^*2*^	0.229	0.232	0.236	0.196	0.204	0.225
F Value	19.637***	23.367***	24.467***	20.873***	21.286***	23.945***
Observations	3227	3227	3227	3227	3227	3227

Note

*, **, and *** indicate that the two-tailed t-test values are statistically significant at the 10%, 5%, and 1% levels, respectively. The year and industry dummies were controlled for all regressions.

Columns (1) to (3) of Table 4 are the results of the impact of state-owned equity in private enterprises on their *ROA*. Specifically, Columns (1) and (2) show that with the company characteristic variables controlled, *SOE* and *SOER* are significantly positive at the level of 1%, indicating that state-owned equity can significantly improve the financial performance of enterprises. In Column (3), the variable of entrepreneurs’ political participation is added, and the results show that *SOE* and *SOER* are significantly positive at the level of 5%, while *Political* is significantly positive at the level of 10%. This demonstrates that the membership in People’s Congress or CPPCC of the actual controller or general manager of private enterprises, combined with the inclusion of state-owned shares, has a significant positive impact on company performance. The last three columns of Table 4 show the impact of state-owned equity in private enterprises on *ROE*, which is consistent with that of *ROA*. Therefore, H1 is supported by the test results in general.

#### 5.2.2 The impact of state-owned equity on enterprises’ access to economic resources

Columns (1) to (4) of Table 5 reveal the impact of state-owned equity on private enterprises’ access to bank loans, and Columns (5) to (8) reveal its impact on private enterprises’ entry into industries with barriers.

**Table 5:** The impact of state-owned equity on enterprises’ access to economic resources.

Variable	*Loan*				*Barrier*			
	(1)	(2)	(3)	(4)	(5)	(6)	(7)	(8)
*SOE*	0.048**	—	0.059**	—	0.134***	—	0.111**	—
	(1.918)		(2.232)		(10.731)		(5.333)	
*SOER*	—	0.034***	—	0.078**	—	0.105***	—	0.124***
		(2.891)		(2.043)		(9.818)		(9.865)
*Tassets*	0.023**	0.055**	0.024	0.018	0.143*	0.095*	0.161	0.263
	(1.745)	(1.831)	(0.851)	(0.787)	(4.839)	(4.663)	(1.966)	(1.041)
*Profit*	0.073***	0.059***	0.058*	0.068	0.071	0.075	0.083	0.064
	(2.888)	(2.908)	(1.469)	(0.987)	(1.921)	(2.018)	(1.853)	(1.523)
*Fassets*	0.046**	0.035**	0.055	0.063**	0.363*	0.541**	0.737	0.053
	(1.696)	(1.651)	(1.024)	(1.771)	(4.721)	(5.228)	(1.516)	(1.735)
*Growth*	0.138*	0.155	0.044	0.021*	0.105	0.127	0.093	0.111
	(1.438)	(0.995)	(1.181)	(1.518)	(1.673)	(1.869)	(1.725)	(0.968)
*Pillar*	0.047**	0.062***	0.096*	0.029*	0.027*	0.038***	0.055*	0.049
	(2.258)	(2.903)	(1.562)	(1.477)	(3.623)	(9.674)	(3.268)	(1.982)
*Cintensity*	0.031	0.019	0.037	0.058	0.027	0.108	0.094	0.065
	(0.617)	(0.738)	(0.520)	(1.091)	(1.468)	(1.573)	(1.488)	(1.577)
*Ltime*	-0.062	-0.134	-0.088	-0.128	-0.037	-0.153	-0.093	-0.115
	(-0.924)	(-0.892)	(-0.765)	(-1.059)	(-1.298)	(-0.916)	(-1.318)	(-1.577)
*Political*	0.082	0.077	0.103	0.083	0.129	0.129	0.108	0.085
	(0.845)	(0.954)	(0.684)	(1.044)	(1.963)	(1.827)	(1.541)	(1.628)
*Tassets×SOE*	—	—	-0.097***	—	—	—	-0.104***	—
			(-3.566)				(-11.764)	
*Tassets×SOER*	—	—	—	-0.107***	—	—	—	-0.128***
				(-2.865)				(-9.329)
Adjusted- *R*^*2*^	0.175	0.207	0.241	0.221	—	—	—	—
F Value	15.259***	18.282***	20.541***	19.343***	—	—	—	—
Nagelkerke- *R*^*2*^	—	—	—	—	0.176	0.198	0.171	0.1866
Percentage Correct	—	—	—	—	79.3	80.4	78.3	81.6
Observations	3227	3227	3227	3227	3227	3227	3227	3227

Note

*, **, and *** indicate that the values are statistically significant at the 10%, 5%, and 1% levels, respectively. The figures in parentheses in Columns (1) to (4) are the t values of the two-tailed tests, and the figures in parentheses in Columns (5) to (8) are the Wald test values. The year and industry dummies were controlled for all regressions.

Columns (1) and (2) of Table 5 concern the impact of state-owned equity on private enterprises’ access to bank loans. Column (1) shows that with the company characteristic variables and the year and industry dummies controlled, the coefficient of *SOE* is significantly positive at the 5% level. The regression results in Column (2) show that the coefficient of *SOER* is significantly positive at the level of 1%, indicating that the proportion of state-owned equity in private-controlled companies has a positive impact on the credit decisions of financial institutions.

To test whether the total assets of enterprises (*Tassets*) and state-owned equity variables (*SOE* and *SOER*) also have substitution effects on enterprises’ ability to obtain economic resources, the interaction terms *Tassets×SOE* and *Tassets×SOER* are introduced in Columns (3) and (4) of Table 5, respectively. The regression results show that the coefficients of *SOE* and *SOER* are significantly positive, that of *Tassets* is positive but not statistically significant, and that of the interaction terms are significantly negative. This suggests that if private enterprises hold state-owned shares or the proportion of state-owned shares is high, financial institutions will significantly reduce the emphasis they placed on enterprise assets during loan decisioning. Columns (5) to (8) of Table 5 concern the effect of state-owned equity on private companies’ entry into industries with barriers. The results show that the presence of state-owned equity has a significant positive impact on private enterprises’ entry into industries with barriers. However, the significant negative coefficients of the interaction terms indicate that if private enterprises have state-owned equity, the role of company assets in entering industries with barriers would be weakened. These test results reveal that the existence of state-owned equity in private-controlled enterprises can enable them to obtain more financial support and development space, so H2 and H3 are verified.

#### 5.2.3 State-owned equity and entrepreneurs’ political participation: The link between the two mechanisms

Table 6 reports the link between the two mechanisms of state-owned equity and entrepreneurs’ political participation.

**Table 6:** The relationship between state-owned equity and entrepreneurs’ political participation.

Variable	*ROA*			*ROE*		
	(1)	(2)	(3)	(4)	(5)	(6)
*SOE*	0.023***	0.033**	—	0.055**	0.031**	—
	(2.027)	(1.741)		(2.317)	(1.885)	
*SOER*	0.019**	—	0.093***	0.067*	—	0.106**
	(2.183)		(2.463)	(1.519)		(1.873)
*Tassets*	0.066	0.028	0.038**	0.084	0.023**	0.069**
	(1.093)	(1.032)	(1.782)	(0.806)	(1.702)	(1.894)
*Leverage*	0.073	0.113	0.0428	0.063	0.048	0.056
	(0.984)	(0.978)	(0.855)	(0.783)	(0.898)	(0.698)
*Growth*	0.049	0.097	0.107	0.108*	0.093	0.128
	(0.838)	(0.836)	(1.082)	(1.509)	(0.873)	(0.984)
*Pillar*	0.027**	0.036*	0.018	0.532**	0.063	0.085
	(2.104)	(1.436)	(0.693)	(1.943)	(0.974)	(1.018)
*Cintensity*	0.017	0.058	0.119	0.062	0.079	0.104
	(0.710)	(0.961)	(0.858)	(0.876)	(0.995)	(0.568)
*Ltime*	-0.048	-0.073	-0.127	-0.095	-0.174	-0.093
	(-0.866)	(-0.973)	(-0.868)	(-0.758)	(-1.125)	(-0.954)
*Political*	0.013*	0.043	0.125	0.063*	0.078	0.103
	(1.492)	(1.212)	(0.977)	(1.504)	(1.054)	(1.145)
*Political×SOE*	—	-0.058***	—	—	-0.108***	—
		(-3.158)			(-2.929)	
*Political×SOER*	—	—	-0.204***	—	—	-0.137***
			(-3.145)			(-2.878)
Adjusted- *R*^*2*^	0.238	0.242	0.214	0.228	0.245	0.237
F Value	24.518***	21.633***	23.608***	23.072***	22.272***	20.639***
Observations	3227	3227	3227	3227	3227	3227

Note

*, **, and *** indicate that the two-tailed t-test values are statistically significant at the 10%, 5%, and 1% levels, respectively. The year and industry dummies were controlled for all regressions.

The above regression results show that state-owned equity and entrepreneurs’ political participation both have a significant positive impact on corporate performance. The regression results of interaction terms *Political×SOE* and *Political×SOE* to *ROA* is introduced in Columns (2) and (3), respectively. We can see that the coefficients of *SOE* and *SOER* are still significantly positive, that of *Political* is positive but not statistically significant, while those of the interaction terms are significantly negative, implying that the impact of entrepreneurs’ participation in politics on enterprise performance would be weakened if the enterprises hold state-owned shares. This reflects that in the current transitional economic system, state-owned equity is a “higher” level of alternative institution, which plays a more crucial role in financing and entering industries with barriers than entrepreneurs’ political participation. Columns (4) to (6) of Table 6 present the regression results to *ROE*, which are similar to those to *ROA*. Moreover, the impact of related control variables is basically consistent with that listed in Table 4, so H4 is validated.

Reforming state-owned enterprises (SOEs) has resulted in the transformation of state-owned enterprises into private enterprises with state-owned shares. For some companies whose original identity was private enterprises, the reason for introducing state-owned equity is complicated. Private enterprises with state-owned shares, whether formed through state-owned reform or the introduction of state-owned shares into private enterprises, hold business and commercial strategies meeting the needs of national development. Therefore, these firms have particularities compared with ordinary private enterprises. In this socio-economic context, the discussion of endogeneity is unnecessary here. It does not mean that outperformance enterprises can introduce state-owned shares according to their wishes. Enterprise strength is not a key and dominant factor. Therefore, there is no advantage for companies with good profits and powerful strength to introduce state-owned shares, and thus they are not more likely to be selected by state-owned enterprises.

### 5.3 Robustness test

To test the reliability of the above regression results, this paper replaced the state-owned equity variables *SOE* and *SOER* with state-owned equity background (*SOEB*) and ratio of state-owned directors on the board (*SOEBR*). The corresponding results are listed in Tables 7–9 respectively. It can be seen that after replacing the relevant indicators depicting the variables of state-owned equity, the new indicators still largely support the hypotheses proposed in this paper. The interaction terms *Fassets×SOE* and *Fassets×SOER* are also constructed in Table 8 to replace *Tassets×SOE* and *Tassets×SOER* to carry out the corresponding regression, and the results are basically in line with the previous analysis. To save space, regression tables are not presented in the paper. The robustness test results validate the high reliability of the empirical results of this study.

**Table 7:** The impact of political connection resulted from state-owned equity on enterprise performance (Robustness test).

Variable	*ROA*			*ROE*		
	(1)	(2)	(3)	(4)	(5)	(6)
*SOEB*	0.073***	—	0.033**	0.028**	—	0.017**
	(3.031)		(1.836)	(2.024)		(1.984)
*SOEBR*	—	0.032***	0.042*	—	0.062**	0.028*
		(3.091)	(1.569)		(1.858)	(1.524)
*Tassets*	0.022	0.019*	0.055	0.048	0.024*	0.018
	(1.019)	(1.493)	(0.727)	(1.003)	(1.483)	(0.886)
*Leverage*	0.108	0.048	0.074	0.043	0.052	0.044
	(0.832)	(0.936)	(0.689)	(0.840)	(0.738)	(0.922)
*Growth*	0.013*	0.109	0.022	0.164	0.108	0.105
	(1.477)	(0.843)	(0.955)	(0.768)	(0.824)	(0.938)
*Pillar*	0.023**	0.028*	0.025*	0.038*	0.045*	0.018*
	(2.018)	(1.596)	(1.584)	(1.494)	(1.284)	(1.492)
*Cintensity*	0.069	0.118	0.098	0.106	0.073	0.059
	(0.997)	(0.759)	(0.965)	(0.753)	(0.872)	(0.974)
*Ltime*	-0.077	-0.065	-0.034	-0.069	-0.031	-0.052
	(-1.032)	(-0.974)	(-0.822)	(-0.684)	(-0.862)	(-0.595)
*Political*	—	—	0.019*	—	—	0.033*
			(1.604)			(1.596)
Adjusted- *R*^*2*^	0.217	0.197	0.235	0.204	0.186	0.224
F Value	22.058***	19.673***	23.155***	21.856***	20.299***	24.036***
Observations	3227	3227	3227	3227	3227	3227

Note

*, **, and *** indicate that the two-tailed t-test values are statistically significant at the 10%, 5%, and 1% levels, respectively. The year and industry dummies were controlled for all regressions.

**Table 8:** Robustness test: The impact of political connection resulted from state-owned equity on enterprises’ access to resources.

Variable	*Loan*				*Barrier*			
	(1)	(2)	(3)	(4)	(5)	(6)	(7)	(8)
*SOEB*	0.025**	—	0.077**	—	0.108***	—	0.127***	—
	(2.042)		(2.168)		(12.082)		(8.366)	
*SOEBR*	—	0.042***	—	0.066**	—	0.245***	—	0.372**
		(3.035)		(1.727)		(9.833)		(5.278)
*Tassets*	0.108*	0.028**	0.093	0.065	0.204**	0.192*	0.168	0.137
	(1.515)	(1.816)	(0.786)	(0.858)	(5.825)	(2.975)	(1.356)	(1.244)
*Profit*	0.063**	0.044***	0.056*	0.073*	0.117	0.088	0.102	0.088*
	(1.658)	(3.205)	(1.614)	(1.422)	(1.867)	(1.684)	(1.593)	(2.537)
*Fassets*	0.014**	0.103*	0.048**	0.062	0.105*	0.295**	0.115	0.210
	(1.886)	(1.603)	(1.933)	(1.127)	(2.972)	(4.717)	(1.315)	(1.466)
*Growth*	0.103*	0.084	0.074*	0.041	0.084	0.105	0.124	0.129*
	(1.504)	(0.963)	(1.643)	(0.764)	(1.688)	(1.284)	(1.277)	(2.763)
*Pillar*	0.091**	0.039*	0.034*	0.032*	0.055*	0.037*	0.052*	0.047
	(2.174)	(1.614)	(1.396)	(1.427)	(2.632)	(2.803)	(2.562)	(1.074)
*Cintensity*	0.104	0.058	0.077	0.008	0.077	0.126	0.105	0.164
	(0.853)	(0.631)	(0.863)	(0.614)	(1.433)	(1.373)	(0.974)	(1.153)
*Ltime*	-0.074	-0.017	-0.084	-0.135	-0.067	-0.105	-0.091	-0.078
	(-0.683)	(-0.875)	(-0.945)	(-1.026)	(-1.762)	(-1.361)	(-1.463)	(-1.211)
*Political*	0.048	0.082	0.024	0.069	0.118	0.095	0.088	0.105
	(1.088)	(0.956)	(1.087)	(1.061)	(1.533)	(1.378)	(1.491)	(1.127)
*Tassets×SOEB*	—	—	-0.106**	—	—	—	-0.197***	—
			(-2.266)				(-8.235)	
*Tassets×SOEBR*	—	—	—	-0.022***	—	—	—	-0.232***
				(-3.053)				(-9.591)
Adjusted- *R*^*2*^	0.207	0.199	0.215	0.239	—	—	—	—
F Value	18.923***	20.856***	21.049***	20.598***	—	—	—	—
Nagelkerke- *R*^*2*^	—	—	—	—	0.181	0.193	0.184	0.207
Percentage Correct	—	—	—	—	81.5	78.9	80.3	82.6
Observations	3227	3227	3227	3227	3227	3227	3227	3227

Note

*, **, and *** indicate that the values are statistically significant at the 10%, 5%, and 1% levels, respectively. The figures in parentheses in Columns (1) to (4) are the t values of the two-tailed tests, and the figures in parentheses in Columns (5) to (8) are the Wald test values. The year and industry dummies were controlled for all regressions.

**Table 9:** Robustness test: The relationship between state-owned equity and the political connection resulted from entrepreneurs’ political participation.

Variable	*ROA*			*ROE*		
	(1)	(2)	(3)	(4)	(5)	(6)
*SOEB*	0.033**	0.028**	—	0.017**	0.108*	—
	(1.838)	(1.946)		(1.986)	(1.629)	
*SOEBR*	0.044*	—	0.138**	0.028*	—	0.084**
	(1.570)		(2.318)	(1.524)		(2.165)
*Tassets*	0.056	0.019	0.022**	0.018	0.054	0.117
	(0.727)	(1.094)	(1.674)	(0.884)	(1.093)	(1.022)
*Leverage*	0.074	0.043	0.036	0.045	0.031	0.034
	(0.689)	(0.927)	(0.734)	(0.921)	(0.768)	(0.829)
*Growth*	0.022	0.124*	0.105	0.104	0.114	0.132
	(0.953)	(1.603)	(0.868)	(0.937)	(1.260)	(1.182)
*Pillar*	0.027*	0.035**	0.036**	0.018*	0.033	0.058
	(1.584)	(1.646)	(2.062)	(1.492)	(0.818)	(0.925)
*Cintensity*	0.098	0.029	0.117	0.059	0.041	0.047
	(0.965)	(0.618)	(0.845)	(0.974)	(0.818)	(0.783)
*Ltime*	-0.034	-0.027	-0.093	-0.052	-0.062	-0.082
	(-0.824)	(-0.982)	(-0.624)	(-0.595)	(-0.693)	(-1.071)
*Political*	0.017*	0.103	0.035	0.033*	0.129	0.077
	(1.602)	(0.597)	(0.916)	(1.596)	(0.848)	(1.193)
*Political×SOEB*	—	-0.013***	—	—	-0.063***	—
		(-3.483)			(-2.834)	
*Political×SOEBR*	—	—	-0.038***	—	—	-0.019***
			(-2.927)			(-3.874)
Adjusted- *R*^*2*^	0.235	0.196	0.225	0.224	0.192	0.215
F Value	23.154***	20.639***	17.525***	24.039***	21.637***	19.327***
Observations	3227	3227	3227	3227	3227	3227

Note

*, **, and *** indicate that the two-tailed t-test values are statistically significant at the 10%, 5%, and 1% levels, respectively. The year and industry dummies were controlled for all regressions.

## 6 Conclusion and policy suggestion

China’s private enterprises have contributed tremendously to China’s economic development, but their development process is difficult and the environment is severely constrained. This is mainly due to the imperfection of relevant laws and systems. This paper explores the role of institutional gaps in informal systems in the development of private enterprises. The concept of ‘alternative institutions’ is the first time to propose and use in corporate finance field research. It provides a clear demarcation for institutions’ impact on corporate finance. Further, the institutional gap is concretized and summarized as introducing state-owned equity in the field of alternative institutions. Therefore, several conclusions we could made as follow. The private enterprises outperformance through introducing state-owned equity compared with those single shareholder private companies. To be explicitly, introducing state-owned equity assist private enterprises in entering industries with high barriers, easily obtain finance support and bank loans. Compared with entrepreneurs’ political participation (non-institutional gap part in alternative institutions), private enterprises introducing state-owned equity exhibit advantages in management performance, enter barrier industry, and gain financial support.

In the current economic transformation background, the presence of state-owned equity in privately held companies undoubtedly plays a role in guaranteeing reputation at the institutional level. The existence of state-owned shareholders reflects that private enterprises become strong. Meanwhile, it provides the signal that they have attracted the favour and support of relevant government departments. It helps enterprises obtain more economic resources. The impact of state-owned equity introduction in private enterprises is reflected in the following: enterprises still need other options to encounter difficulties and gain development, not only rely on state-owned equity to obtain many benefits in terms of financing convenience and entering industries regulated by the government. The related laws and regulations to protect the development of private enterprises need to be further improved. Private enterprises directly use political power to reduce economic losses, or even use administrative means through the government to ensure the smooth operation of the enterprise. Moreover, the political connection effect brought about by state-owned equity is based on the institutional level, and its role should exceed the political connection effect of private entrepreneurs participating in politics at the superficial level. Therefore, there is reason to believe that the introduction of state-owned equity is a very important channel for private enterprises.

The purpose of the research is also to provide a theoretical and empirical reference for government departments to design systems for mixed ownership reform. The research results of this article have important implications and policy implications. First of all, in the process of economic transformation, the government should deepen the reform of the economic system. Only when the business environment of enterprises continues to improve, can enterprises more consciously rely on market mechanisms to allocate resources efficiently and rationally, instead of actively seeking connections with the government to obtain benefits. In addition, in the operation process of state-owned equity participation in private enterprises, the corporate governance level of state-owned equity should be improved. The supervisory role of checks and balances shareholders should be exerted to the greatest extent to maintain and increase the value of state-owned assets, rather than allowing the controlling private shareholders to use them as resources acquired political capital.

## Supporting information

S1 File (DOCX)
