# Supplementary material for: Institutional gap and enterprise behavior: Evidence from China
Source: PLoS One. 2024 Mar 1;19(3):e0297455. doi: 10.1371/journal.pone.0297455 (PMC10906870; doi:10.1371/journal.pone.0297455)
Supplement: S1 File — (DOCX) [file pone.0297455.s001.docx]

JOURNAL REQUIREMENTS:

1. Please ensure that the author list and affiliations are correct on the title page of your manuscript, and that your author contributions, competing interests, and financial disclosure are correct as listed below. All of these sections will be indexed in PubMed and published by PLOS ONE as you have written them. Please email [plosone@plos.org](mailto:plosone@plos.org) if any changes to this content needs to be made.

Author Contributions: conceived and designed the experiments: Meijie Yao, Wenxue Wang, Li Rao
Author Contributions: performed the experiments: Meijie Yao
Author Contributions: analyzed the data: Meijie Yao, Wenxue Wang
Author Contributions: contributed reagents/materials/analysis tools: Meijie Yao, Wenxue Wang, Li Rao
Author Contributions: wrote the manuscript: Meijie Yao, Wenxue Wang
Author Contributions: other:

Competing Interests: We declare that we have no known competing financial interests or personal relationships that could have appeared to influence the work reported in this paper.

Financial Disclosure: This study was supported by fundamental research funds for the central universities (Grant numbers. 2023CDSKXYJG007)

2. Please confirm that all information in your Funding Information is also present in your Financial Disclosure. Only the Financial Disclosure section will be published alongside your article to describe your funding.

Funding Information: This study was supported by fundamental research funds for the central universities (Grant numbers. 2023CDSKXYJG007).
